# Keeping good nursing records: a guide

**Published:** 2010-12

**Authors:** Sue Stevens, Dianne Pickering

**Affiliations:** Former Nurse Advisor, Community Eye Health Journal, International Centre for Eye Health, London School of Hygiene and Tropical Medicine, Keppel Street, London WC1E 7HT, UK.; Nurse Advisor, Community Eye Health Journal; Registered General Nurse, Norfolk and Norwich University Hospital, UK. **dianne.pickering@nnuh.nhs.uk**

**Figure:**
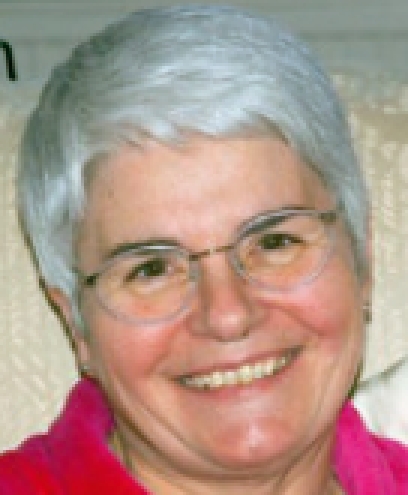


**Figure:**
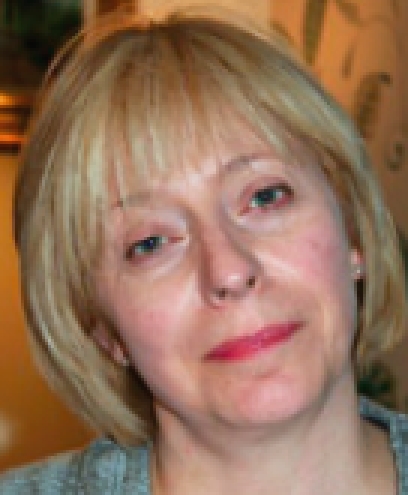


In the busy working day of a nurse, with the many urgent demands on your time, you may feel that keeping nursing records is a distraction from the real work of nursing: looking after your patients.

This cannot be more wrong! Keeping good records is part of the nursing care we give to our patients. It is nearly impossible to remember everything you did and everything that happened on a shift. Without clear and accurate nursing records for each patient, our handover to the next team of nurses will be incomplete. Needless to say, this can affect the wellbeing of patients.

In fact, the quality of our record keeping can be a good (or bad) reflection of the standard of care we give to our patients: careful, neat, and accurate patient records are the hallmarks of a caring and responsible nurse, but poorly written records can lead to doubts about the quality of a nurse's work.

Another important consideration is the legal significance of nursing records. If a patient brings a complaint, your nursing records are the only proof that you have fulfilled your duty of care to the patient. According to the law in many countries, if care or treatment due to a patient is not recorded, it can be assumed that it has not happened. Poor record keeping can therefore mean you are found negligent, even if you are sure you provided the correct care - and this may cause you to lose your right to practise.

In short, the patient's nursing record provides a correct account of the treatment and care given and allows for good communication between you and your colleagues in the eye care team. Keeping good nursing records also allows us to identify problems that have arisen and the action taken to rectify them.

**‘Keeping good nursing records allows us to identify problems that have arisen and the action taken to rectify them’**

In this article, we discuss how to be effective in your record keeping and how to maintain the high standards required.

## Who is responsible for record keeping?

Anyone on the nursing team who provides patient care can contribute to record keeping. However, if you are a qualified or senior nurse supervising unqualified colleagues, you should assume responsibility for providing guidance on documentation.

## What should go into a patient's nursing record?

The nursing record is where we write down what nursing care the patient receives and the patient's response to this, as well as any other events or factors which may affect the patient's wellbeing. These ‘events or factors’ can range from a visit by the patient's relatives to going to theatre for a scheduled operation.

If you are in any doubt about what to write down, it may be useful to ask yourself the following: “If I was unable to give a verbal handover to the next nursing team, or the next shift, what would they need to know in order to continue to care for my patients?” You want to ensure that the patient's care is not affected by the changeover of nursing staff.

## How to keep good nursing records

The patient's record must provide an accurate, current, objective, comprehensive, but concise, account of his/her stay in hospital. Traditionally, nursing records are hand-written. Do not assume that electronic record keeping is necessary.

- Use a standardised form. This will help to ensure consistency and improve the quality of the written record. There should be a systematic approach to providing nursing care (the nursing process) and this should be documented consistently. The nursing record should include assessment, planning, implementation, and evaluation of care.
- Ensure the record begins with an identification sheet. This contains the patient's personal data: name, age, address, next of kin, carer, and so on. All continuation sheets must show the full name of the patient.
- Ensure a supply of continuation sheets is available.
- Date and sign each entry, giving your full name. Give the time, using the 24-hour clock system. For example, write 14:00 instead of 2 pm.
- Write in dark ink (preferably black ink), never in pencil, and keep records out of direct sunlight. This will help to ensure they do not fade and cannot be erased.
- On admission, record the patient's visual acuity, blood pressure, pulse, temperature, and respiration, as well as the results of any tests.
- State the diagnosis clearly, as well as any other problem the patient is currently experiencing.
- Record all medication given to the patient and sign the prescription sheet.
- Record all relevant observations in the patient's nursing record, as well as on any charts, e.g., blood pressure charts or intraocular pressure phasing charts. File the charts in the medical notes when the patient is discharged.
- Ensure that the consent form for surgery, signed clearly by the patient, is included in the patient's records.
- Include a nursing checklist to ensure the patient is prepared for any scheduled surgery.
- Note all plans made for the patient's discharge, e.g., whether the patient or carer is competent at instilling the prescribed eye drops and whether they understand details of follow-up appointments.

**Figure:**
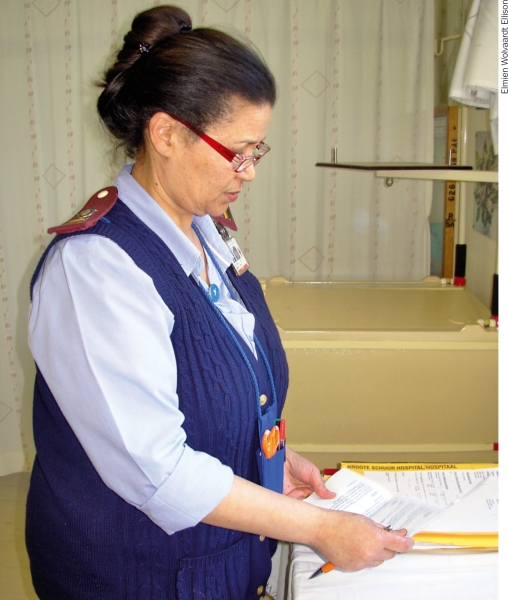
Patients' records must provide an accurate account of their hospital stay. SOUTH AFRICA

Elmien Wolvaardt Ellison

## Writing tips

- Ensure the statements are factual and recorded in consecutive order, as they happen. Only record what you, as the nurse, see, hear, or do.
- Do not use jargon, meaningless phrases, or personal opinions (e.g., “the patient's vision appears blurred” or “the patient's vision appears to be improving”). If you want to make a comment about changes in the patient's vision, check the visual acuity and record it.
- Do not use an abbreviation unless you are sure that it is commonly understood and in general use. For example, BP and VA are in general use and would be safe to use on records when commenting on blood pressure and visual acuity, respectively.
- Do not speculate, make offensive statements, or use humour about the patient. Patients have the right to see their records!
- If you make an error, cross it out with one clear line through it, and sign. Do not use sticky labels or correction fluid.
- Write legibly and in clear, short sentences.
- Remember, some information you have been given by the patient may be confidential. Think carefully and decide whether it is necessary to record it in writing where anyone may be able to read it; all members of the eye care team, and also the patient and relatives, have a right to access nursing records.

## Looking after nursing records

Keep the nursing records in a place where they can be accessed easily; preferably near to where the nursing team meet at shift change times. This will ensure that records are available for handover sessions and also that they are easily accessible to the rest of the eye care team. The handover may take place with the patient present, if appropriate. Indeed, nursing records can only be accurate if patients have been involved in decision making related to their care.

File the nursing records in the medical notes folder on discharge. Ensure that the whole team knows if nursing records are stored elsewhere.

## How can nursing records contribute to VISION 2020?

Accurate records will contain observations of clinical outcomes, for example, how an elderly patient has benefited from his or her cataract operation or how skilled the patient is at instilling eye drops before discharge. Such information can be used in clinical audit and reports on clinical activity. This contributes to research and performance data which can be used to monitor improvement in service delivery and outcomes, all of which ultimately contributes to VISION 2020. It is not only medical notes that are important; well-written nursing records will provide qualitative comment on treatment outcomes.

